# Individual cognitive remediation therapy benefits for patients with anorexia nervosa and high autistic features

**DOI:** 10.1002/erv.2707

**Published:** 2019-11-11

**Authors:** Yasemin Dandil, Katherine Smith, James Adamson, Kate Tchanturia

**Affiliations:** ^1^ Department of Psychological Medicine, Institute of Psychiatry, Psychology Neuroscience King's College London London UK; ^2^ South London and Maudsley NHS Foundation Trust Eating Disorders National Service London UK; ^3^ Department of Psychology Illia State University Tbilisi Georgia

**Keywords:** adults, anorexia nervosa, autism spectrum disorders, cognitive remediation, comorbidity

## Abstract

Cognitive remediation therapy (CRT) is an increasingly implemented intervention in psychiatric conditions. The majority of randomized treatment trials in psychiatry reports cognitive improvements resulting in better functional outcomes in CRT groups. This brief report from the national inpatient treatment programme for eating disorders demonstrates cognitive performance task–based improvements in patients with high and low autistic characteristics. This preliminary study shows feasibility and benefits of individual CRT in patients who have autism spectrum disorder features.

## 1 INTRODUCTION

Cognitive remediation therapy (CRT) aims to target inefficiencies in bigger picture thinking and cognitive flexibility known to be cognitive maintaining factors for anorexia nervosa (AN; Tchanturia, Giombini, Leppanen, & Kinnaird, 2017; Tchanturia, Lounes, & Holttum, 2014). CRT is available in individual (eight to 10) sessions and group formats (five to six) sessions for patients with AN (Tchanturia, Davies, Reeder, & Wykes, 2010). The positive effects of CRT on patients with AN have been demonstrated in both naturalistic studies and randomized control trials (Leppanen, Adamson, & Tchanturia, 2018; Sproch, Anderson, Sherman, Crawford, & Brandt, 2019).

Inefficiencies in cognitive flexibility (set‐shifting) and poor Gestalt processing (central coherence) are also commonly seen in individuals with autism spectrum disorders (ASDs; Westwood, Stahl, Mandy, & Tchanturia, 2016). A feasibility study looking at individual CRT for patients with ASD has found improvements in central coherence but not for set‐shifting (Okuda et al., 2017). Interestingly, there is thought to be a higher prevalence of ASD in patients with AN (Westwood & Tchanturia, 2017). Despite the high comorbidity, there has been little research into the way clinical symptoms of ASD impact AN treatments. Previous research has suggested that the presence of high ASD traits in patients with AN reduces the effectiveness of group CRT when compared with those with low ASD traits (Tchanturia, Larsson, & Adamson, 2016). To date, no research has looked at the effects of individual CRT on patients with comorbid ASD features and AN. Therefore, this naturalistic study aimed to examine the difference in the effects of individual CRT treatment for adult women with AN on an inpatient treatment programme with either high‐ or low‐comorbid ASD characteristics.

## 2 METHODS

### 2.1 Participants

Participants consisted of 99 females admitted to the inpatient treatment programme in the specialist national eating disorder service. The inclusion criteria for the study were adult females with a diagnosis of AN (either subtype) according to the Diagnostic and Statistical Manual of Mental Disorders (Fifth Edition, DSM‐5; American Psychiatric Association, 2013), with completed Autism Spectrum Quotient (AQ‐10) scores and approaching their first course of individual CRT. Only participants who took part in individual CRT and agreed to complete the neurocognitive measures preintervention were included in the study. Fifty‐nine participants met the threshold for high ASD characteristics using the AQ‐10, and the remaining 40 participants did not meet the clinical cut‐off for ASD. The individual CRT format consists of eight to 10 sessions (duration of 30–40 min) shortly after the admission, typically within the first 2 weeks. The patients mean (*M*) age was 23.9 years, standard deviation (*SD*) of 6.2, and the mean body mass index was 14.3 (*SD* = 1.3) at Time 1. At Time 2, postintervention, the mean body mass index was 15.3 (*SD* = 1.4).

### 2.2 Procedure

All participants underwent individual CRT as described in Tchanturia et al. (2010). Participants completed neurocognitive measures before and after the intervention as well as AQ‐10 on admission. Participants were drawn from a previously analysed data set (Leppanen et al., 2018); however, for the purposes of this study, participants were divided according to their scores on the AQ‐10, to differentiate ASD traits. We examined the interaction of AQ‐10 scores with individual CRT treatment.

### 2.3 Measures

Both thinking styles were assessed with one performance‐based measure and one subcategory of the self‐reported questionnaire. The following neurocognitive features were assessed: central coherence (“bigger picture thinking”), assessed with the performance‐based Rey–Osterrieth Complex Figure (ROCF) test (Rey, 1941) and later standardized by Osterrieth (Osterrieth, 1944) for large data set (Lang et al., 2016), set‐shifting measured with the performance‐based Brixton Spatial Anticipation Test (Burgess & Shallice, 1996) for large data set (Tchanturia et al., 2011), cognitive rigidity, and attention to detail measured with the self‐reported Detail and Flexibility (DFlex) Questionnaire (Roberts, Barthel, Lopez, Tchanturia, & Treasure, 2011).

The Autism Spectrum Quotient, short version (AQ‐10), is a brief, validated measure of autistic traits, showing equal validity to the extended version of the AQ (Allison, Auyeung, & Baron‐Cohen, 2012). Patients completed the AQ‐10 on admission, and using a clinical cut‐off of 6 with higher scores indicating increased expression of ASD traits, patients were grouped into high and low ASD traits.

### 2.4 Statistical analysis

To assess the impact of AQ‐10 on changes on neuropsychological and self‐reported cognitive outcomes, we used a linear mixed‐effects analysis with AQ‐10 included as a covariate. Linear mixed‐effects analyses were conducted using R (R Development Core Team, 2013); the effect of time (baseline scores compared with end of treatment) was entered as a fixed‐effect predictor. Neurocognitive assessments (Brixton and ROCF) scores were available from 99 and 98 participants, respectively, at baseline and 61 and 59 at the end of treatment. Self‐reported, DFlex scores were available from 54 participants at baseline and 34 participants at the end of treatment. A secondary analysis was conducted using participants AQ‐10 scores with 6 as a clinical meaningful cut‐off of high and low scorers. Low scorers were compared with high scorers using repeated measures ANOVA on Brixton and ROCF scores, before and after CRT. Main effects of time and group were computed using repeated measures ANOVA and independent samples *t* tests. DFlex was not analysed in this way due to the small sample size in each subgroup. Secondary analysis and database management was conducted using IBM's SPSS Statistics Version 24 (IBM, 2016).

### 2.5 Ethics

The study was approved by the hospital clinical governance committee.

## 3 RESULTS

### 3.1 Effect of AQ‐10 on CRT outcomes

The results from the linear mixed‐effects analysis suggest that AQ‐10 scores did not have a significant effect on change in Brixton scores over time, *F*(148) = 0.036, *p* > .05. Furthermore, AQ‐10 scores did not affect the relationship of DFlex, attention to detail subscale, and scores over time, *F*(54) = 26.94, *p* > .05. However, there was a small significant effect of AQ‐10 scores on ROCF outcomes, *F*(136) = 3.93, *p* < .05, suggesting that AQ‐10 scores may influence the relationship between baseline and end of treatment ROCF scores. Furthermore, a significant effect of AQ‐10 on DFlex, cognitive rigidity subscale, was found, *F*(57) = 10.45, *p* < .01.

### 3.2 Secondary analysis

**Figure 1:**
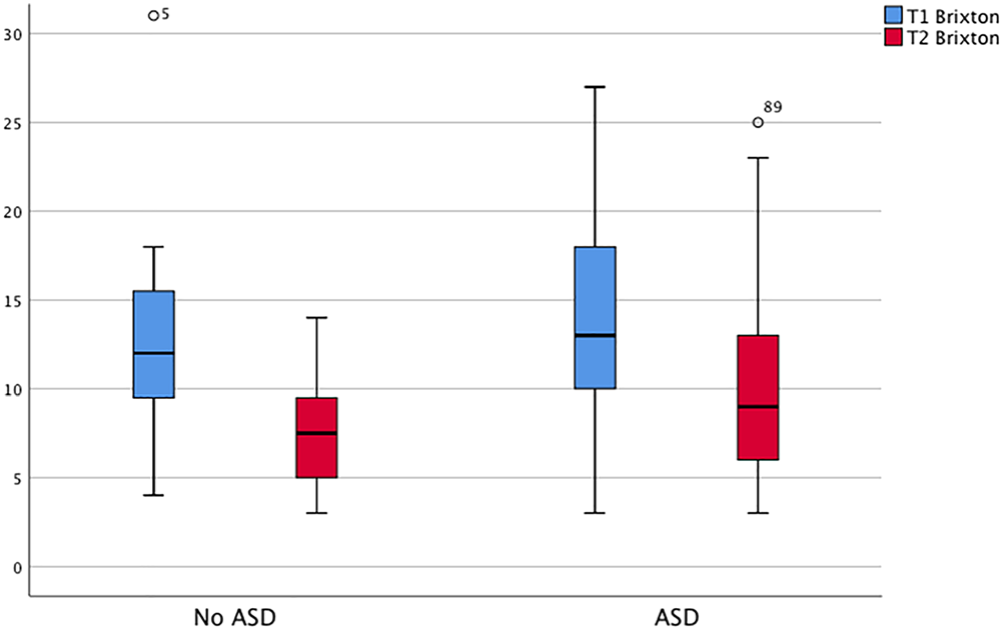
Brixton score comparison at the start and end of cognitive remediation therapy split into those that score under and over cut‐off on the AQ‐10. ASD, autism spectrum disorder [Colour figure can be viewed at http://wileyonlinelibrary.com]

Paired‐sample results for the 61 particpants with both premeasure and postmeasure indicate that there was a significant main effect of time on both Brixton scores, *t*(60) = 8.57, *p* < .001, and ROCF central coherence, *t*(58) = −2.35, *p* < .05, with both improving over time. For those with complete data, 36 participants (59%) scored below cut‐off on the AQ‐10, and 25 participants scored above (41%). There were no significant differences between those that scored above or below cut‐off on baseline Brixton scores, *t*(97) = 0.02, *p* > .05, or ROCF central cohernace scores, *t*(96) = 0.52, *p* > .05. Those that scored below cut‐off showed a significant increase in Brixton scores over the course of CRT, *t*(35) = 6.74, *p* < .001, with a mean and standard deviation at the start of treatment of 12.72 (4.78) improving to 7.83 (3.31) at the end of treatment. Those that scored above cut‐off on the AQ‐10 also showed significant improvements in Brixton scores, *t*(24) = 5.3, *p* < .001, with a mean at the start of treatment of 14.28 (6.38) improving to 10.36 (5.76) after CRT. Results for Brixton are displayed in Figure 1.

**Figure 2:**
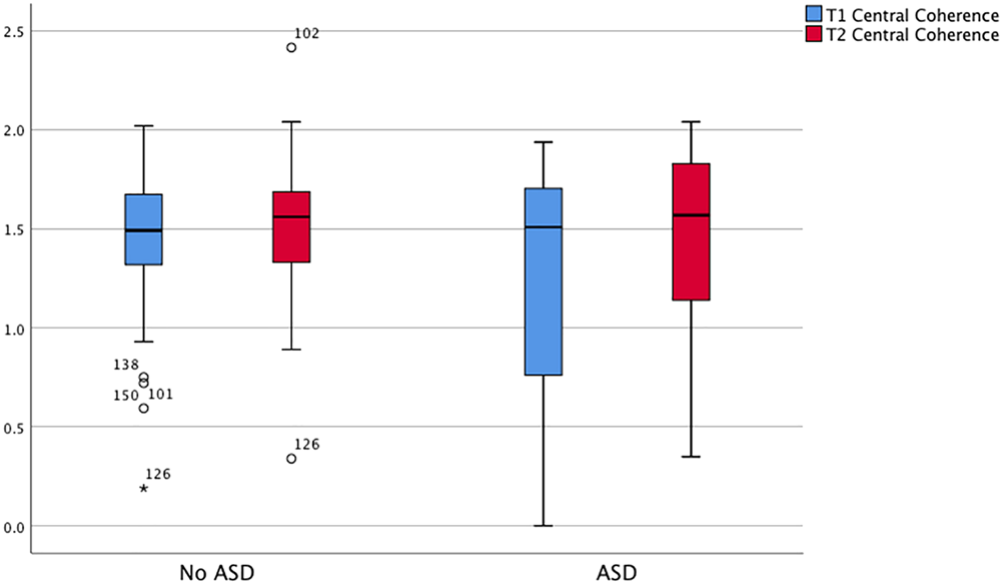
Rey–Osterrieth Complex Figure (ROCF) test central coherence scores at the start and end of cognitive remediation therapy split into those that score under and over cut‐off on the AQ‐10. ASD, autism spectrum disorder [Colour figure can be viewed at http://wileyonlinelibrary.com]

However, those that scored below cut‐off did not significantly improve in central coherence on the ROCF, *t*(34) = −1.44, *p* > .05, with a mean difference (*MD*) between start and end of CRT of −0.09 (0.38). On the other hand, although those that scored above cut‐off also did not see a statistically significant improvement, *t*(23) = −1.9, *p* = .06, they did see a larger *MD* in central coherence of −0.15 (0.38). Results for the ROCF are displayed in Figure 2.

## 4 DISCUSSION

In this brief naturalistic study, we have explored cognitive test performances before and after individual CRT in patients with high and low ASD characteristics receiving inpatient treatment for AN. We also conducted self‐reported measures on the same aspects of cognition: flexibility and bigger picture thinking.

Our results from this naturalistic observational study demonstrated that having high ASD features had no effect on scores in neurocognitive measures of flexibility using the Brixton Test or the self‐reported measures of attention to detail measured with the DFlex (Roberts et al., 2011). However, differences were found based on AQ‐10 scores for neurocognitive performances in bigger picture thinking measured with the ROCF (Rey, 1941; cited in Osterrieth, 1944) and self‐reported cognitive rigidity using the DFlex (Roberts et al., 2011).

The results regarding the Brixton Test were in line with previous findings of positive improvements posttreatment in both groups (Leppanen et al., 2018). This suggests that the presence of high ASD traits does not impact the effects of individual CRT. Looking at the secondary analysis, we can also see that there is an improvement in mean scores for cognitive flexibility for both groups. However, the ASD group starts at a more severe point, resulting the ASD group to still appear high at the end but not relatively to Time 1 scores (Tchanturia, Adamson, Leppanen, & Westwood, 2019).

No effect difference was seen of AQ‐10 scores on the self‐reported attention to detail subscale of the DFlex. Due to the small sample sizes of the DFlex, we could not run a secondary analysis. Therefore, it would be interesting to look at this in future studies.

Small significant effects were seen in the ROCF outcomes based on ASD traits (AQ‐10 scores). Furthermore, our secondary analysis found that there was no significant improvement based on the ROCF scores in central coherence for either group, which goes against previous research of this data set (Leppanen et al., 2018). The differences here could be due to our analysis only being conducted on patients with available AQ‐10 scores, which has excluded 46 participants from the original data. From Figure 2, we can see that there is a much larger spread of data in the high ASD trait group; the sample size was very small at 23, hence the large standard deviation. This could be improved with a larger sample size.

A further significant effect of the AQ‐10 was found on the self‐reported cognitive rigidity (DFlex—cognitive rigidity subscale). This is interesting considering that the performance‐based measure for cognitive rigidity (the Brixton Test) was not significant. This inconsistency between performance‐based tasks and self‐reported measures is also found between the measures of bigger picture thinking (ROCF and DFlex—attention to detail). In support of this finding, a study conducted by Lounes, Khan, and Tchanturia (2011) highlighted that there is poor correspondence between the self‐report measure of cognitive flexibility and performance on the flexibility task; this suggests that the different measures tap into different aspects of cognitive processing. It would be beneficial to explore which test measures which aspect of cognitive processing in order to best support clinical application.

To our knowledge, this is the first report comparing individual CRT data between patients with AN with high and low ASD features. The naturalistic study reported in this paper has many limitations (e.g., no randomization, missing self‐reported data in some cases, and brief AQ‐10 measure). However, we believe that this report is still valuable for researchers as feasibility evidence. Future studies would benefit from larger sample sizes, specifically in the self‐reported DFlex and in the high ASD trait groups as well as interview‐based ASD diagnostic measures.

In addition to the interesting research aspect, we think that this study provides some preliminary evidence of the feasibility of supporting patients with severe and enduring AN, which have ASD comorbidity or high levels of ASD features, and may benefit from treatment adaptations. The benefits we have reported in this study suggest that more evidence generating work in the area will be worthwhile.
